# A Nonenzymatic and Automated Closed-Cycle Process for the Isolation of Mesenchymal Stromal Cells in Drug Delivery Applications

**DOI:** 10.1155/2018/4098140

**Published:** 2018-02-20

**Authors:** Valentina Coccè, Anna Brini, Aldo Bruno Giannì, Valeria Sordi, Angiola Berenzi, Giulio Alessandri, Carlo Tremolada, Silvia Versari, Antonio Bosetto, Augusto Pessina

**Affiliations:** ^1^CRC StaMeTec, Department of Biomedical, Surgical and Dental Sciences, University of Milan, Milan, Italy; ^2^I.R.C.C.S Galeazzi Orthopedic Institute, Milan, Italy; ^3^Maxillofacial and Dental Unit, Fondazione IRCCS Cà Granda Ospedale Maggiore Policlinico, Via Commenda 10, 20122 Milan, Italy; ^4^Diabetes Research Institute, IRCCS San Raffaele Scientific Institute, Milan, Italy; ^5^University Research Center “Integrated Models for Prevention and Protection in Environmental and Occupational Health” (MISTRAL), Brescia, Italy; ^6^Cellular Neurobiology Laboratory, Department of Cerebrovascular Diseases, Fondazione IRCCS Neurological Institute C. Besta, Milan, Italy; ^7^Image Institute, Milan, Italy; ^8^Lipogems International Spa, Milan, Italy; ^9^Hydra srl, Mirandola, Modena, Italy

## Abstract

The adipose tissue is a good source of mesenchymal stromal cells that requires minimally invasive isolation procedures. To ensure reproducibility, efficacy, and safety for clinical uses, these procedures have to be in compliant with good manufacturing practices. Techniques for harvesting and processing human adipose tissue have rapidly evolved in the last years, and Lipogems® represents an innovative approach to obtain microfragmented adipose tissue in a short time, without expansion and/or enzymatic treatment. The aim of this study was to assess the presence of mesenchymal stromal cells in the drain bag of the device by using a prototype Lipogems processor to wash the lipoaspirate in standardized condition. We found that, besides oil and blood residues, the drain bag contained single isolated cells easy to expand and with the typical characteristics of mesenchymal stromal cells that can be loaded with paclitaxel to use for drug-delivery application. Our findings suggest the possibility to replace the drain bag with a “cell culture chamber” obtaining a new integrated device that, without enzymatic treatment, can isolate and expand mesenchymal stromal cells in one step with high good manufacturing practices compliance. This system could be used to obtain mesenchymal stromal cells for regenerative purposes and for drug delivery.

## 1. Introduction

Mesenchymal stromal cells (MSCs) can be easily isolated from several human organs and tissues and, because of their self-renewing capacity and multipotent differentiation properties, they are important tools for treating immune disorder and for tissue repair. In particular, adipose-derived MSCs (ASCs) can be harvested in high amounts by minimally invasive procedures with good viability, differentiating potential, and paracrine activity [1–3]. These cells can be also used as drug carrier (as already demonstrated for MSCs derived from bone marrow) because, when primed with high doses of the chemotherapeutic drug paclitaxel (PTX), they are capable to uptake the drug and release it in big amounts inhibiting the *in vitro* proliferation of different tumour cell lines [4, 5]. Their definition as advanced-therapy medicinal products (ATMPs) according to the European Medicines Agency (EMA) and the US Food and Drug Administration requirements for their production and use implies the application of production processes that should be in accordance with good manufacturing practices (GMPs). To increase safety and reproducibility and to implement the many clinical trials using ASCs, several methods based on reducing the manipulation of human tissue-based products have been suggested, in particular by using nonenzymatic treatments [6, 7]. Among the many, an innovative closed and sterile system (named Lipogems), designed to harvest, process, and transfer a refined and not-expanded adipose tissue characterized by a great regenerative potential and optimal handling, has been developed. By the aid of this technology, and without the addition of enzymes or any other additives, fat tissue is microfragmented in a completely close liquid environment and washed from proinflammatory oil and blood residues [8]. This closed and sterile device reduces the size of the adipose tissue clusters by means of two filters [9]. A drain bag, connected to the second filter of the device, collects the waste fluid containing red blood cells and oil residues. We used a prototype Lipogems processor (PLG-P) to wash and process the lipoaspirate in a standardized condition and we found that, besides oil and blood residues, the drain bag contained single isolated cells easy to expand and with the typical characteristics of ASCs. These cells can be also loaded with paclitaxel to provide a cell-mediated drug-delivering tool.

## 2. Materials and Methods

### 2.1. Ethics Statements

Samples from adult donors were collected after signed informed consent of no objection for the use for research of surgical tissues (otherwise destined for destruction) in accordance with the Declaration of Helsinki. The approval for their use was obtained from the Institutional Ethical Committee of Milan University (n.59/15, C.E.UNIMI, 09.1115).

### 2.2. Prototype Lipogems Processor (PLG-P)

**Figure 1:**
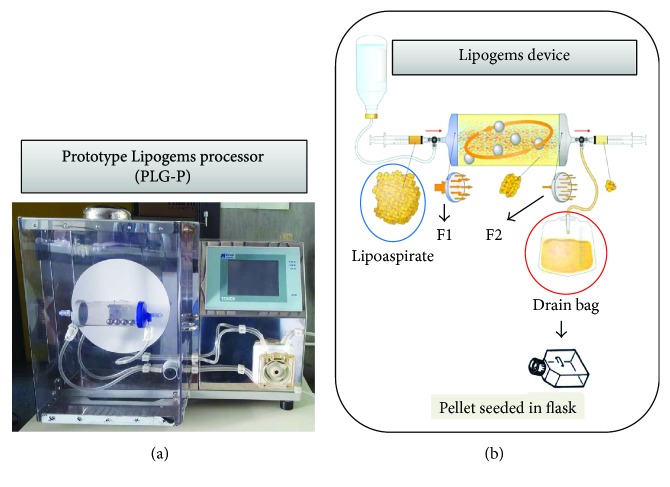
DB-MSC isolation and expansion. (a) Prototype Lipogems processor (PLG-P) mounting a Lipogems device. In this closed system, the lipoaspirate is microfragmented by using mild mechanical forces without the addition of collagenase or other enzymes/additives. (b) Schematic representation of Lipogems device. The lipoaspirate (blue circle) is subjected to a cluster reduction by pushing the fat into the device through the first filter (F1). LGP-P shaking transmits a mechanical force to the stainless steel marbles contained in the device that results into a temporary fat emulsion washed by a flux of buffer. This allows the washing buffer to cross a second filter (F2) and accumulate into the drain bag (red circle) together with oil, red blood cells, and mesenchymal stromal cells (ASCs). The pellet obtained after centrifugation was seeded in 25 cm^2^ flask in StemMACS medium (Miltenyi Biotec, USA) (see Materials and Methods for details).

The prototype processor (Figure 1(a)) equipped with the Lipogems device (Figure 1(b)) is able to guarantee digitally controlled movements according to these main parameters: oscillation amplitude and frequency and pitch movements and saline washing flux. 25 ml of lipoaspirate was loaded in the Lipogems device and washed according to a standardized procedure: process flow 120 ml/min, oscillation frequency 2 Hz, pitch frequency 0.3 Hz, pitch angle 30°, and pitch axis 90° vertical (exit bottom). In a first preliminary experiment, the tissue was processed for 5 minutes and cells were collected one step into 600 ml of saline. In a second experiment, the collection was fractioned and performed at 1 (120 ml), 2.5 (180 ml), and 5 minutes (300 ml).

### 2.3. ASC Isolation from Drain Bag (DB-ASCs)

100 ml of each sample was centrifuged at 2500 ×g for 15 minutes. Pellet was treated twice with 0.85% ammonium chloride (5 minutes at +4°C) to partially remove red cells. Then, after three washes with PBS 1x through centrifugation (800 ×g, 10 minutes), pellet was plated on 25cm^2^ flask (Corning, USA) with 5 ml of Stem MACS MSC Expansion Medium (Miltenyi Biotec, Germany) and incubated at 37°C, 5% CO_2_. The primary culture was expanded with 1 : 2 passages until passage number (pn) 6. At pn 2, the population doubling time (PDT) was calculated as previously described [10].

The clonogenicity of the ASCs was evaluated as colony-forming efficiency (CFE) in multiwell plates (SPL Life Sciences, Korea) by serial dilution (from 50 cells/well to 1 cell/well) in DMEM LG media with 10% fetal bovine serum (EuroClone, UK). After 10 days, cell colonies fixed and labeled with Crystal Violet (0.5%, Fluka, Switzerland) were counted at the microscope (a colony formed by at least 25 cells) and the CFE as the following: CFE = average of colonies formed × 100/number of seeded cells.

### 2.4. Immunohistochemical Analysis of DB-MSCs

Mesenchymal cell monolayer (pn 2) was detached with trypsin-EDTA (EuroClone, UK) and 1.7^∗^10^6^ cells were placed in a 10 ml glass conical tube in 1x PBS and centrifuged at 800 ×g for 10 minutes. Pellet was treated for immunohistochemical characterization as previously reported [11]. Briefly, the centrifuged pellet was treated 20 minutes at room temperature with a solution of 75% methanol, 20% chloroform, and 5% glacial acetic acid. The pellet compacted in a solid mass was placed in an immunohistochemical cassette and immersed in a 10% buffered formaldehyde solution and, after dehydration in ethanol and paraffin fixation, the pellet was cut in sections of 4-5 *μ*m that were stored at −20°C. For phenotypic characterization, monoclonal or polyclonal antibodies were used using the streptavidin-biotin method with diaminobenzidine as chromogen. The following antibodies were used: antismooth muscle actin (SMA), anti-CD14, anti-CD105, and anti-neural/glial antigen 2 (NG2) (Santa Cruz Biotechnology, USA); anti-CD44 (Monosan, Holland); antivascular endothelial growth factor (VEGF), anti-CD90 (Dako, Italy); anti-CD45, anti-CD146, anti-CD31, and anti-CD34 (Leica Biosystems, Germany).

The expression of CD73 (CD73-PE Becton Dickinson, USA) was evaluated by flow cytometry (FacsVantage SE, Becton Dickinson, USA). Cells were washed twice in PBS, diluted to a final concentration of 10^6^ cells/ml, and incubated with the dark antibody at +4°C for 30 minutes. The cells were then washed with PBS and analysed by cytofluorometry by acquisition of 10,000 events per sample. The results were analysed with CellQuest Pro software (Becton Dickinson, USA).

### 2.5. DB-ASC Osteo-/Adipo-Differentiation Capacity

DB-ASCs at pn 2 have been evaluated for their osteogenic and adipogenic differentiation using a standardized procedure [12]. As positive control, MSCs obtained from human bone marrow (BM-MSCs) were isolated and expanded in our laboratory as previously reported [5]. As negative control, MSCs were cultured without the addition of supplements. The differentiation was performed by plating cells in 35 mm Petri (Nunc, Germany) at a density of 50 cells/cm^2^ in 1 ml of STEM MACS MSC Expansion Medium (Miltenyi Biotec, Germany). After 72 hours of incubation, the cell medium was removed and replaced with a specific medium for cell differentiation. To induce osteogenic differentiation [13], cells were incubated for 14 days in DMEM medium LG + 20% fetal bovine serum in the presence of the following: desametasone 10 nM, glycerol 2–10 mM, and phosphate and ascorbic acid 300 nM (all reagents, Sigma-Aldrich, USA). The differentiation medium was replaced every 3-4 days with fresh medium. Cell monolayer was fixed with cold methanol for 5 minutes at −20°C, then alkaline phosphatase (ALP) was evaluated by SIGMA FAST BCIP/NTB substrate for ALP (Sigma-Aldrich, USA). The colorimetric reaction was detected by optical microscope. The presence of osteoblasts at the mature stage was evaluated by staining with Alizarin Red S (Sigma-Aldrich, USA). To induce adipocytic differentiation, cells were cultured in DMEM LG + 20% fetal bovine serum in the presence of indomethacin 200 *μ*M (Alexis Biochemicals, USA), isobutylmethylxanthine 0.5 mM (AppliChem, Germany), dexamethasone 1 *μ*M, hydrocortisone 1 *μ*M insulin, and 10 *μ*g/ml (all from Sigma-Aldrich, USA). After 10 days of incubation, the cells were fixed with 10% buffered formalin (Sigma-Aldrich, USA) for 30 minutes at room temperature and the adipocyte vacuoles present in the cell cytoplasm were evaluated by using red lipophilic Oil Red O (Sigma-Aldrich, USA).

### 2.6. Ability of DB-ASCs to Uptake and Release Paclitaxel

The ability to incorporate and release drugs from DB-ASCs has been assessed with a procedure already applied on MSCs obtained from bone marrow, adipose tissue, human gingival tissue, human fibroblasts, and also blood cells [4, 5, 10, 14–16]. The “uptake-release” method was performed using paclitaxel (PTX) that is an anticancer drug with also antiangiogenic activity. Subconfluent ASC cultures (15,000 cells/cm^2^) were exposed to 2000 ng/ml of PTX (Fresenius Kabi, Italy) dissolved in culture media, and after 24 hours of incubation (37°C, 5% CO_2_), the cell monolayer was washed with PBS and the cells detached, washed by centrifugation in PBS, and subcultured in a 25 cm^2^ flask with fresh culture media. After 48 hours, the conditioned medium (CM) was collected and its antitumour activity was evaluated *in vitro*. The CM from untreated cells was used as a negative control.

### 2.7. Cytokines in DB-ASC Secretome

The conditioned medium of DB-ASCs (from untreated or treated PTX cells) was collected, and the cytokine content was evaluated using “multiplex bead-based xMAP technology” (Bio-Plex Human Cytokine 27-Plex Panel and Bio-Plex Human Group II Cytokine 21-Plex Panel, Bio-Rad Laboratories).

### 2.8. *In Vitro* Antiproliferative Assay on CFPAC-1 of CM from PTX-Loaded MSCs

The effect of pure paclitaxel and CM from PTX-treated ASCs (CM/PTX) against tumour cell proliferation has been studied in 96-multiwell plates (Sarstedt, Germany) by using as target pancreatic adenocarcinoma cells (CFPAC-1) [17]. Briefly, 1 : 2 serial dilutions of pure drug or ASC-CM were prepared in 100 *μ*l of culture medium/well and then to each well were added 1000 tumour cells. After 7 days of culture at 37°C and 5% CO_2_, cell growth was evaluated by MTT assay (3-(4,5-dimethyl-2-thiazolyl)-2,5-diphenyl-2-H-tetrazolium) as previously described [5, 18]. The inhibitory concentrations (IC_50_ and IC_90_) were determined according to the Reed and Muench formula [19]. The antitumor activity of PTX CM was compared to that of pure PTX and expressed as PTX equivalent concentration: PEC (ng/ml) = IC50 PTX × 100/V50 (*μ*l/well); IC50 PTX = the concentration of pure PTX producing 50% of inhibition; V50 is the volume PTX-MSCs-CM able to inhibit cell proliferation by 50%. The PEC referred to a single primed ASC was calculated as ratio between the total amount of PEC (PEC (ng/ml) × CM volume (ml)) and the number of cells seeded: PE release (pg/cell) = PEC (ng tot)^∗^1000/number of cells seeded. Uptake release experiments were performed in triplicate.

### 2.9. Statistical Analysis

Experiments were performed using lipoaspirate from three donors and repeated in triplicate. The reported data are expressed as mean ± standard deviation. If necessary, appropriate statistical tests have been performed using GraphPad Software (GraphPad Inc., San Diego, CA, USA). A *p* value ≤ 0.05 was considered statistically significant. The linearity of response and the correlation were studied using regression analysis, by Excel 2007 software (Microsoft Inc.).

## 3. Results

### 3.1. DB-ASC Isolation and Expansion

**Figure 2:**
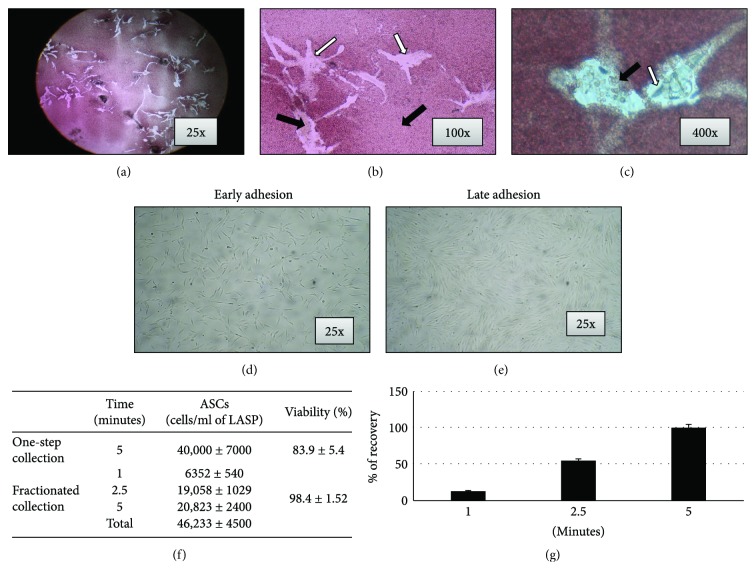
Growth and recovery efficiency of DB-ASCs. After few days of culture, it was possible to distinguish the red blood cells from the ASCs adherent to the flask (a, b, c). In Figures (b) and (c), black arrows indicate red blood cells and white arrows ASCs. Figure (d) represents the early adhesion (10 days at 37°C, 5% CO_2_), and Figure (e) the late adhesion (15 days at 37°C, 5% CO_2_) of DB-ASCs. Figure (f) reports DB-ASC recovery efficiency (the amount of ASCs recovered from the lipoaspirate by one-step collection and by fractionated collection), and the histogram (g) reports the percentage recovery of ASCs by fractionated collection after 1, 2.5, and 5 minutes. Data are expressed as mean ± standard deviation (SD) of three different measures.

The waste medium recovered from the drain bag contained high amounts of red cells that were removed, only partially, by the ammonium chloride treatment. However, after about 7 days, the adhered ASCs could be clearly identified, by transparency, surrounded by a compact rug of red cells (Figures 2(a)–2(c)). However, the adherent cells (washed every 3 days) improve their growth and after 10 days (early adhesion) or 15 days (late adhesion) of incubation, the cell monolayer (Figures 2(d) and 2(e)) was detached and primary culture (pn 0) was quantified for the number of cells. The MSC primary culture was subsequently expanded by 1 : 2 serial passage until passage number 6. MSC growth was evaluated at pn 2 by evaluating the population doubling Time (PDT) and the colony-forming efficiency (CFE) that resulted, respectively, 55.2 ± 2.3 hours (PDT) and 20.8% ± 10.4% (CFE).

### 3.2. DB-ASC Recovery Efficiency

To evaluate the amount of ASCs recovered from the lipoaspirate, the washing fluid was collected from the drain bag by two different ways. In a first experiments (one-step collection), 600 ml of saline was collected after 5 minutes of washing; in a second experiment (fractionated collection), the collection was performed at 1 minute (120 ml), 2.5 (180 ml), and 5 minutes (300 ml) (Box 2F). In the one-step collection, the total recovery resulted 40,000 ± 7000 ASCs/ml of lipoaspirate with a cell viability of 83.9 ± 5.4% as evaluated by trypan blue assay. By fractionated collection, the final recovery at 5 minutes was 46,233 ± 4500 ASCs/ml with a viability of 98.4 ± 1.52. As shown, ASCs accumulate into the drain bag according to a kinetic that collects 13.7% of cells at 1 minute, 54.9% at 2.5 minutes and, at the end of the process, the recovery (100%) is similar to what was observed in the one-step procedure but with a higher percentage of cell viability (Figure 2(g)).

### 3.3. Immunohistochemical Analysis of DB-ASCs

**Figure 3:**
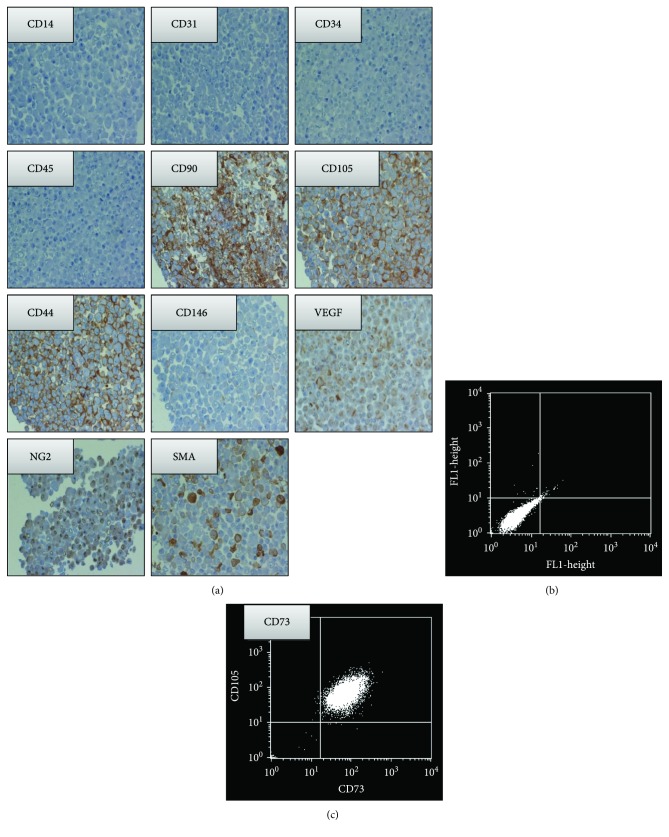
Immunohistochemical analysis of DB-ASCs. (a) Immunohistochemical analysis for CD14, CD31, CD34, CD45, CD90, CD105, CD44, CD146, NG2, SMA, and VEGF (all figures are 100x magnification). (b, c) FACS analysis on CD73: (b) = negative CTRL and (c) = CD73 expression.

The immunohistochemistry analysis performed on ASCs showed the high positivity for CD44, CD90, and CD105 (>90%) and the negativity for CD45, CD34, CD31, and CD14 hematopoietic markers (Figure 3(a)). The positivity for CD73 was evaluated by FACS and expressed by the dot-blots (Figure 3(c)). Significant was also the expression of SMA (cytoplasmic and membrane) and VEGF (cytoplasmic). Only a small percentage of cells are positive for CD146, whereas the presence of reactivity for NG2 seems to be mainly expressed at the nuclear level (Figure 3(a)).

### 3.4. DB-ASC Osteo-/Adipo-Differentiation Capability

**Figure 4:**
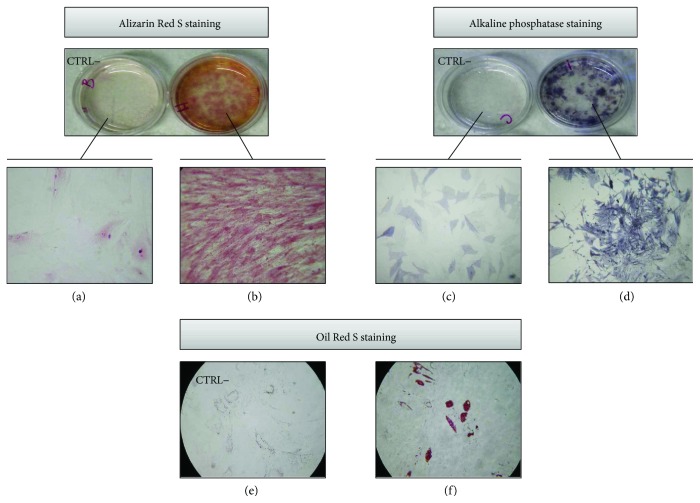
DB-ASC osteo-/adipo-differentiation. Osteogenic differentiation evaluated in Petri dish by Alizarin Red S staining (left) and by phosphatase alkaline staining (right). (a, c) DB-ASCs grown in control medium without supplements (negative control, CTRL−); (b, d) positive culture (200x magnification). Adipogenic differentiation evaluated by Oil Red staining (presence of red cytoplasmic inclusions; 200x magnification). (e) Negative control (CTRL−) (200x magnification). (f) Positive culture (200x magnification).

The differentiation capacity towards osteogenic and adipogenic lineages is reported in Figure 4.

A remarkable positivity of ALP (Figure 4(d)) confirms the presence of a significant enzymatic activity involved in the mineralization of the bone matrix. The mature stage of osteoblasts is confirmed by the presence of calcium deposits forming complex orange-colored bifurcations, evidenced by the coloration with Alizarin Red S (Figure 4(b)). As shown in Figure 4(f), DB-ASCs are also able to differentiate in adipocytes as evidenced by the red intracellular inclusions. The negative controls (DB-ASCs cultured without the addition of supplements) do not give any evidence of differentiation (Figures 4(a), 4(c), and 4(e)).

### 3.5. Ability of DB-ASCs to Uptake and Release Paclitaxel

**Figure 5:**
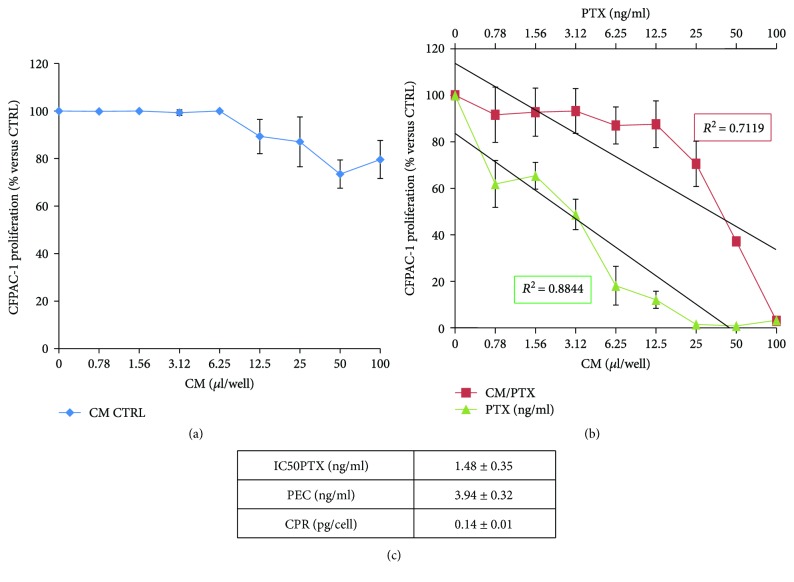
Ability of DB-ASCs to uptake and release paclitaxel. The graph reports the inhibitory activity exerted on pancreatic carcinoma CFPAC-1 cells by (a) the conditioned medium (CM) of nontreated cells (CM CTRL) and (b) the CM of DB-ASCs loaded with PTX (CM/PTX). The activity is compared to the inhibition exerted by pure PTX. The activity is expressed as percentage of CFPAC-1 proliferation referred to that of untreated cells (100% proliferation). The regression and the correlation coefficient (*R*^2^) of the dose-response kinetics are also reported. The box (c) reports the IC_50_ value referred to PTX (ng/ml), the paclitaxel equivalent concentration (PEC), and the single cell paclitaxel release (CPR). See details in Materials and Methods. Values are expressed as mean ± standard deviation (SD) of three independent experiments.

The conditioned media from DB-ASCs, both primed with PTX (CM/PTX) or not (CM CTRL), were tested on the standard laboratory tumour cell line CFPAC-1 (Figure 5). As expected, the CM CTRL does not inhibit neoplastic growth, which remains stable to 80–100% of proliferation (Figure 5(a)). On the contrary, CM/PTX affected CFPAC-1 cell growth according to a dose-dependent antiproliferative effect that, even if less effective, reflects the inhibition produced by free PTX (from 0.39 to 50 ng/ml). The inhibitory kinetics analysed by linear regression showed high coefficients of determination (*R*^2^) ranging from 0.71 to 0.88 (Figure 5(b)). The biological dosage of the CM/PTX based on the standard dose-response regression of the pure drugs enabled the estimation of an equivalent paclitaxel concentration (PEC) of 3.94 ± 0.32 ng/ml. The amount of PTX released by a single cell (CPR), expressed as pg/cell, was 0.14 ± 0.01 pg/cell (box 5C). The drug concentrations released by 10^6^ DB-ASCs/PTX were 140 ng of PTX that correspond to values of about 10 times the IC_50_ value of the free drug (1.48 ± 0.35 ng/ml).

### 3.6. Cytokines in DB-ASC Secretome

**Figure 6:**
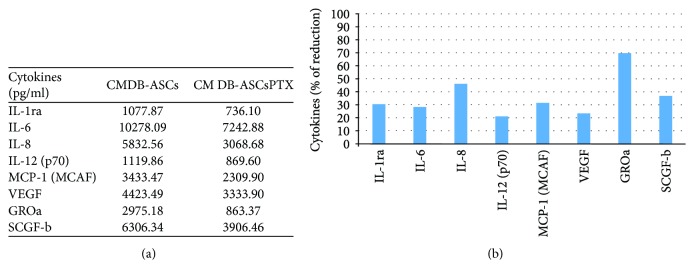
Cytokines in DB-ASC secretome. (a) The table shows the amounts of cytokines (expressed in pg/ml) secreted in the conditioned medium of DB-ASCs before (CM DB-ASCs) and after treatment with PTX (CM DB-ASCs PTX). (b) The histogram reports the decrease of cytokines secretion by PTX-treated DB-ASCs expressed as percentage of the amount produced by the untreated DB-ASCs.

Among the 40 cytokines analysed in the conditioned medium of DB-ASCs primed or not with PTX (CM DB-ASCs; CM DB-ASCs PTX), only those whose secretion was greater than 1000 pg/ml were considered (IL-1ra, IL-6, IL-8, IL-12 (p70), MCP-1 (MCAF), VEGF, and GROa) and reported in Figure 6. The concentration of these cytokines in the CM was evaluated before and after PTX treatment. As it was shown, the PTX treatment seems to inhibit their secretion that is expressed as percentage ranged from 22.35% (IL12 (p70)) to 70.98% (GROa) with a mean decrease of 37.1 ± 15.7%.

## 4. Discussion

It is known that adipose tissue can be harvested by minimally invasive procedures according to different methodologies and many efforts have been done to reduce the manipulation of the biological material [6]. Lipogems represents a very interesting technique that, without the addition of enzymes or any other additives, microfragments the lipoaspirate in a closed liquid environment that allows, by the protection provided by the liquid (which is noncompressible by definition), to avoid the destruction of the adipocytes. The final product is a nonexpanded and minimally manipulated adipose tissue product suitable for clinical uses because it is in compliant with good manufacturing practices (GMPs). As recently reviewed, Lipogems has been used in many surgical fields such as orthopaedic, reconstructive, and plastic surgery and oncology [20]. In order to obtain more standardized microfragmenting conditions, a prototype apparatus (PLG-P) has been designed that overcoming the manual operations provides a procedure with programmable parameters (angle and frequency of shaking and flux of washing). We mounted a Lipogems device into the PLG-P equipped with a drain bag allowing the collection of high volumes of the washing fluid object of our investigations (Figure 1). Our study demonstrated that the “waste” material contained in the drain bag is surprisingly a rich source of ASCs. In fact, during the long microfragmentation process, the continuous shaking and washing flux allows to detach mechanically many ASCs that accumulate into the drain bag. Of course, the significant amount of single isolated ASCs is diluted into the bag due to the extensive washing volume of buffer used. However, these cells can be easily collected by centrifugation and if cultured, in the presence of contaminant red cells, are able to adhere with a significant CFE and can be easily expanded with a good PDT (Figure 2). These cells are positive for the expression of CD44, CD73, CD90, CD105, SMA, VEGF, and NG2 and negative for CD14, CD31, CD34, and CD45 and showed osteo-/adipogenic differentiation capability (Figures 3 and 4). Although it is known that the expression of these markers may depend on culture conditions, the pattern clearly confirm the mesenchymal stromal cell type of DB-ASCs. Besides the positivity for the typical MSC markers CD90, CD105, and CD73[21], DB-ASCs are also positive for NG2 (about 50%) and SMA (90%) that are markers commonly expressed by pericytes in different amount depending from the type of vessel from which they originated (capillaries, venules, or arterioles) [22, 23]. Lipoaspirate is very rich in microvessels, and, therefore, a significant amount of ASC progenitors can be obtained with our procedure. Very few cells were positive for CD146, probably because the expression of this marker is reduced in the ASC population washed from lipoaspirate, in agreement with our previous study on MSCs in Lipogems suggesting that CD146 could stain mostly cells of endothelial origin rather than pericytes [24].

The possibility to estimate the cell content and, in particular, the number of ASCs/ml of lipoaspirate give importance to this technique. Our study suggests that washing the lipoaspirate with the PLG-P allows to collect significant amounts of single isolated ASCs with a recovery efficiency, in terms of ASCs per ml of lipoaspirate processed, of 46,233 ± 5500 ASCs/ml that is higher compared to what was reported by other authors [25].

A further important observation is that the expanded ASCs were able to uptake and then release paclitaxel (PTX). Although the PTX treatment modified the amount of some cytokines produced by DB-ASCs (Figure 6), this aspect is not relevant because cells loaded with the drug are able to release PTX in an active form as shown by the *in vitro* anticancer activity of the conditioned medium (Figure 5). The uptake-release ability of paclitaxel by DB-MSCs is in line with our previous observations on MSCs from different sources (bone marrow, adipose tissue, human gingival tissue, human fibroblasts, and also blood cells) and confirms that these cells can be considered an important tool for cell-mediated drug delivery [4, 5, 10, 14–16].

## 5. Conclusions

In conclusion, this system minimally manipulate the adipose tissue thus bypassing the complex requirements of GMP guidelines, with a dramatic reduction of the costs for cell-based therapies on human patients. From a biotechnological point of view, our findings suggest the possible development of a new integrated device that operating without enzymes allows the isolation, expansion, and drug loading of ASCs in one single step. If equipped with selective filters and if the drain bag will be replaced by a “cell culture chamber,” the PLG-P + Lipogems device configures a “standardized automated collection system” to isolate mesenchymal cells with a “minimal manipulation” compliant with the GMP standards [26, 27]. A system as such has the potential to obtain MSCs not only for regenerative medicine purposes but also for cell-mediated drug delivery.
